# Welfare costs of suboptimal retiree decisions

**DOI:** 10.1371/journal.pone.0307379

**Published:** 2024-08-27

**Authors:** Harun Aydilek, Asiye Aydilek

**Affiliations:** 1 College of Integral Studies, Abdullah Al Salem University, Khaldiya, Kuwait; 2 College of Business and Entrepreneurship, Abdullah Al Salem University, Khaldiya, Kuwait; University of Pisa, ITALY

## Abstract

We make a novel investigation of welfare costs associated with various suboptimal decisions made by retirees, both analytically and numerically. We utilize a unique framework that incorporates recursive utility with housing, and also encompasses expected utility and recursive utility without housing as special cases. Our findings indicate that under-investment in stocks incurs lower welfare costs compared to an equivalent over-investment. Suboptimal allocations in bond holdings result in higher costs than similar misallocations in stocks. Choosing not to participate in the stock market is less detrimental than avoiding the bond market. Should retirees opt to simplify their decision-making by investing solely in one type of asset, it is less costly for them to invest exclusively in bonds. Overconsumption of housing is less costly than an equivalent underconsumption. Suboptimal consumption imposes the highest welfare cost. Decisions regarding consumption, housing, and savings are found to be more crucial than the choice of how to distribute liquid savings between stocks and bonds. Additionally, recursive utility model better captures retirees’ preference for bonds over stocks than expected utility model. Our results, which are consistent across various parameter settings, provide valuable insights for academics and policymakers aiming to enhance retiree welfare.

## 1 Introduction

Standard or traditional economic theory posits that individuals make rational decisions that align with their clear and consistent preferences, aiming to maximize their utility. A decision is deemed ‘rational’ if it represents the optimal choice under given circumstances. However, it is widely acknowledged in both public discourse and interdisciplinary research that people often do not make optimal decisions, suggesting deviations from theoretical rational behavior.

An empirical finding is categorized as an anomaly or suboptimal when it contradicts established economic principles, or when it requires implausible assumptions for explanation. Notably, households often engage in behaviors such as excessive trading, overconfidence, and under-diversification, which counter the notion of economic rationality in human decision-making.

Suboptimal decisions refer to choices that do not maximize one’s well-being. For example, at an ice cream shop with a wide selection of flavors, if a boy selects a flavor that he merely likes instead of choosing his favorite flavor, which is also available, that’s a suboptimal decision. He made a choice, but it wasn’t the most beneficial one for his personal satisfaction.

When individuals fail to choose the best option available, they are committing economic or financial mistakes. These consumer behaviors are challenging to reconcile with models of optimal decision-making (as discussed in studies like [1]. Numerous academic studies have investigated the range of bad economic/financial errors that households make. For example, when it comes to credit card financing, several studies have found that borrowers frequently don’t make their loan payments on time, which results in exorbitant costs [2–4]. Additionally, people often do not diversify their investment portfolios adequately and generally save too little [5]. Furthermore, research indicates that a considerable number of individuals completely avoid engaging in stock market trading [6, 7].

[8] highlights significant challenges faced by households in managing their finances effectively. [9] document that some households simultaneously hold high-interest credit card debt and low-interest checking balances. [10, 11] find that households often miss opportunities for tax efficiency by improperly allocating heavily taxed assets in taxable accounts and lightly taxed assets in tax-deferred accounts. These findings suggest a gap between ideal financial management practices and the real actions taken by households.

Suboptimal decisions often stem from the intricate nature of personal financial planning, limited financial literacy, constraints on time, cognitive biases, or restricted mental capacity. Backwards induction is a possible solution to the underlying optimization problem, yet many scholars, including [12, 13], contend that individuals typically lack the capability to execute such complex computations. Decisions that deviate from rationality frequently involve cognitive biases and heuristics ([14–16] proposed heuristics for their optimization problems.)—these are mental shortcuts or “rules of thumb” that people use to simplify decision-making in the face of overwhelming information.

The term “welfare cost” refers to any decrease in overall happiness or satisfaction resulting from economic decisions. Our primary objective is to measure the welfare costs associated with various suboptimal decisions. We do not delve into the reasons behind retirees’ suboptimal decision-making.

One example of suboptimal decisions is the intriguing observation that many individuals opt not to engage in stock market investments. This is the so-called ‘Stock Market (non) Participation Puzzle’(*SMPP*) and explored by [17]. In other words, the “stock market non-participation puzzle” refers to the phenomenon where many people, even those who can afford to invest, choose not to participate in the stock market. This is perplexing to economists because investing in stocks is typically viewed as a viable strategy for long-term wealth accumulation. [18], utilizing data from the Panel Study of Income Dynamics (*PSID*), find that 72.4% of households in their sample held no stocks at all. This contradicts with the theoretical models which predict that all rational individuals would do so (e.g. [19]).

The observed pattern of limited stock market participation could be another instance of suboptimal decisions. Theoretical models, such as those proposed by [20], suggest that those who do invest in the stock market should allocate a significant portion of their assets to equities. During the prosperous 1990s, when U.S. stock markets saw considerable growth [21], mentions that stock market participation remained limited. Similarly [22], find that only 52% of U.S. households owned stocks in 2004. Additionally [23], report that only 24.2% of households held wealth in stocks or mutual funds in 2007. Moreover, *PSID* data reveals that the median U.S. household has zero direct or indirect risky asset holdings, underscoring the widespread nature of cautious or conservative investment behaviors.

[24], using *AHEAD* data, find that the elderly generally do not tap into their housing equity for current consumption unless faced with significant life changes such as the death of a spouse. Similarly, [25], basing her findings on the Retirement History Survey (*RHS*), find that there is minimal reduction in housing equity among families as they age, unless there are changes in family status. This behavior is at odds with traditional life-cycle theory, which posits that utility-maximizing individuals build up wealth early in life and draw it down in later years [26]. [27] notes that elderly homeowners tend to over-consume housing, maintaining residences larger than their needs, a phenomenon referred to as the ‘housing puzzle’. The ‘housing puzzle’ of the elderly is an another example of suboptimal allocations.

We examine the welfare costs of the puzzling decisions mentioned above as well as other suboptimal cases. We find a modest (3.7%) welfare cost for not holding stocks. This low cost can be regarded as negligible or zero if we also factor in rare-disaster cost of equities, transaction and complexity costs [28, 29]. However, our findings indicate a higher welfare cost, nearly 7%, for not holding bonds. This suggests that for retirees considering investments in either stocks or bonds, it is better to invest in bonds.

As another key finding, our analysis reveals that suboptimal bond investments are costlier than suboptimal stock investments. To exemplify, the utility cost of investing only 20% of the optimal level is about 2.4% for stocks and approximately 4.5% for bonds. Nonetheless, these costs are relatively small; for example, utility costs for deviations up to 100% over optimal stock investments remain below 3.8%. Based on these findings, it appears that issues like stock market non-participation or limited stock investments may not warrant significant concern from policymakers or researchers from a welfare perspective.

Our findings highlight high and asymmetric costs associated with suboptimal housing choices. Specifically, under-consuming housing incurs greater costs compared to over-consuming. For instance, to offset a 90% shortfall from the optimal housing level, a compensation of 34% is required, whereas only an 8% adjustment is needed for an equivalent overage. This suggests that the economic impact of under-consumption in housing is more severe than that of over-consumption.

Our study reveals that the highest welfare costs arise from suboptimal consumption levels. For example, consuming only 30% of the optimal amount results in a substantial utility loss of 41%, whereas investing merely 30% of the optimal amount in bonds leads to a significantly lower utility cost of 3.5%. Consequently, we determine that the most crucial household decisions, from a welfare standpoint, involve consumption, housing, and saving. The specific allocation of liquid savings, however, does not significantly impact overall welfare. Therefore, our research holds significant policy implications for the elderly, which are detailed in Section 8.

It is commonly understood that decisions which might appear irrational or suboptimal could potentially be justified by specific individual preferences. In essence, any decision, regardless of how unconventional it may seem, could be rationalized by identifying a utility function where that choice yields the greatest benefit. Nonetheless, for a utility function to be both practical and credible, it must not only resonate with general behavioral intuitions but also be underpinned by robust empirical evidence. Thus, having testable assumptions is particularly valuable.

Our choice of suboptimal decisions may need more clarification. For example, we categorize stock market non-participation as suboptimal because it is at odds with textbook financial theory. According to textbook financial theory, regardless of their risk aversion level, all households should hold some stocks if there is a positive equity premium (The equity premium is the extra return that investors expected to earn from investing in stocks compared to safer investments like bonds). Similarly, we classify low stock holdings as suboptimal; neoclassical portfolio choice theories indicate that, given existing equity premiums, households should maintain a higher proportion of stocks. Additionally, we identify the ‘housing puzzle’—where elderly individuals do not downsize their homes—as suboptimal. This is based on behavioral expectations that retirees require less space after their children move out and standard life-cycle models that anticipate a reduction in housing consumption among the elderly. Essentially, our analysis focuses on decisions that diverge from well-established theoretical models, economic theories, or expected behavioral norms.

We employ a stylized model that deliberately omits various frictions in both stock and housing markets to concentrate on fundamental dynamics. We also overlook the complexities of decision-making and financial planning to maintain a focus on the core mechanisms. While these simplifications are intentional, it is valid to question whether our conclusions would stand if these real-world complexities were factored in. We posit that including these factors would likely reinforce our findings and perhaps provide stronger support, as discussed in the Model section. This approach suggests that our results are robust and might be even more persuasive if examined within a more comprehensive model.

The welfare analysis of retirees is crucial as we witness a global demographic shift towards an aging population. It is projected that the number of individuals aged 60 and older will double by 2050, with many from the baby boomer generation already entering retirement. This demographic shift has profound implications for saving, consumption, and housing trends. Despite the high poverty rates observed among the elderly, they often remain overlooked in policy making. By understanding the welfare costs associated with different suboptimal retiree decisions, policy makers can develop more effective pension systems and public policies. Tailoring these strategies specifically to the needs of the elderly could significantly enhance their welfare. Some policy suggestions are provided in Section 8.

## 2 Motivation

Suppose a person has a flawless recipe for his favorite dish. Adhering precisely to this recipe should yield the best taste, representing the maximum welfare or happiness from the dish. If the person decides to omit an ingredient or change the amounts—akin to suboptimal resource allocation—the dish may still be enjoyable, but it won’t achieve the highest satisfaction possible. This reduction in satisfaction illustrates the cost of not achieving the optimal culinary outcome, analogous to a loss in welfare or utility.

In economic terms, the “best recipe” equates to the optimal combination of consumption, housing, and investments in stocks and bonds. Altering this mix can lead to a “financial meal” that may not deliver the highest level of satisfaction or welfare. The critical question then becomes: what is the impact of deviating from this optimal allocation? Specifically, how does the exclusion of an asset from a portfolio influence a household’s overall satisfaction or utility? Our research presents a comprehensive framework to explore these questions, which is crucial for a variety of reasons.

Firstly, substantial losses in utility from not adhering to the ideal portfolio composition might warrant more frequent adjustments, or rebalancing, to return to the optimal mix despite potential costs such as fees or taxes. This research aims to determine whether it is more beneficial to modify allocations—akin to adjusting a recipe—or to just leave it as is.

Secondly, using valuable insights from the welfare costs, policymakers, researchers, and financial advisors can pinpoint which suboptimal decisions most adversely affect retirees’ well-being. Figuring out which financial mistakes hurt retirees the most, they can prioritize their advice, policies, and tools to correct those first, thereby making a more pronounced impact. In other words, experts can create a suite of tools—akin to financial repair kits—that tackle costly problems and help retirees manage their money better.

Thirdly, quantifying the financial losses incurred by retirees due to suboptimal decisions can serve as a wake-up call, alerting them to the consequences of less-than-ideal choices. By highlighting the potential pitfalls of certain decisions, such as inadequate savings or poor investment choices, retirees may be motivated to enhance their financial literacy and make more informed decisions.

Fourthly, increased public awareness of how specific decisions can lead to financial challenges fosters greater support for initiatives aimed at enhancing financial literacy. This awareness drives the creation of better educational tools. When everyone understands the common mistakes and their impacts, there can be a community-wide push to improve resources and support for retirees, ultimately ensuring a more seamless journey for them.

Finally, wealth accumulation usually reaches its highest point in the initial years of retirement over a person’s lifetime. Consequently, the financial decisions of older individuals greatly impact their financial security during the asset depletion phase. Suboptimal decisions, such as excessive investment in high-risk stocks or overspending on non-essential luxuries, can rapidly deplete resources. Ultimately, by quantifying the welfare costs associated with suboptimal decisions, we contribute to enhancing the overall retirement security and quality of life for retirees.

In conclusion, our analysis addresses a critical issue that is essential for crafting policies, programs, and educational efforts aimed at enhancing retirees’ quality of life. By fostering financial stability and ensuring sustained consumption levels over time, these initiatives have the potential to significantly improve retirees’ well-being.

## 3 Contribution to the literature

Most research in economics and finance uses standard utility (*SU*) models although recursive utility (*RU*) model is more general and flexible. Our work draws inspiration from previous works like [30–32] that use *RU*, although they don’t explore suboptimal allocations like we do.

A large number of macroeconomic and finance models [30, 33–40] use *RU*. As also mentioned by [41], *RU* disentangles the relative risk aversion (*RRA*) and the willingness to substitute consumption over time or intertemporal elasticity of substitution (*EIS*)—factors that are usually intertwined in *SU* models. The separation of the *EIS* and *RRA* is achieved by imposing a timing attitude on the household, which either prefers early or late resolution of uncertainty. We also model preferences with *RU*. Since *RU* includes *SU* as a special case, we compare the implications of *RU* and those of *SU*, adding strength to our approach.

[42] show that disentangling risk aversion from the *EIS* generates new economic insights. They find that the person with a low wealth and higher *EIS* consumes less as she is more willing to decrease her current consumption for higher consumption in the future, ceteris paribus. [43] discuss some advantages of *RU* while building dynamic stochastic general equilibrium models. Suboptimal decisions may significantly reduce people’s welfare, as highlighted by several studies. [44] suggest that employees may experience losses in utility due to not optimizing their investment and savings choices effectively. [45] use Swedish data to quantify the welfare losses that result from suboptimal portfolio choices. Similarly [46], find substantial losses in life-time utility due to heuristic decision rules.

[47] develop a model showing that investors who ignore market sentiment (Market sentiment is how people collectively feel about a particular market’s direction. When the sentiment is positive, people are generally optimistic and expect prices to go up, leading to more buying.) can achieve higher expected returns, despite incurring a slight mental cost. This mental cost, even as minor as 0.001% of consumption, can lead to significant market sentiment effects. They find that such sentiment behaves like a tragedy of the commons, where individual rational actions lead to collectively suboptimal outcomes.

One strength of our model is that we obtain the analytical solution of the welfare costs, hence pinpointing the exact welfare losses in certain cases. In other cases, where an analytical solution isn’t feasible, we use recursive numerical methods to achieve highly accurate results. This is a significant improvement over most existing literature, which often lacks analytical outcomes. Our ability to solve these problems analytically offers valuable insights into retiree decision-making and the associated welfare costs under suboptimal conditions. Our analytical solution of retiree decisions and welfare costs can serve as a foundation for future research that may explore various scenarios using different parameters.

Changing the utility function is like changing a major building block. However, exploring different types of utility functions and comparing their implications is fruitful and enriching for the literature. Our sensitivity analysis shows that most of our results do not depend on the parameter values assumed and the utility specification. Another strength of our paper is that the choice of utility function is not driving our results. This strength demonstrates that our methodological contribution is sound and significant, providing reliable insights regardless of the utility function used.

While several studies using *RU* models overlook housing, our research incorporates it separately due to its significance for most U.S. households. This inclusion allows us to provide a more comprehensive and realistic analysis of suboptimal allocations. Although [33] also model housing in *RU* framework, they do not explore suboptimal allocations. Thus, our study not only adds depth to the understanding of housing’s role in personal finance but also expands on existing literature by exploring the implications of not optimally utilizing this key asset.

*RU* without housing is also a special case of our setting. Our setting not only enables us to examine *RU* with housing, but also allows us to contrast it with scenarios where housing is not considered. Such comparisons are vital as they help us understand how explicitly including housing affects other financial decisions and overall welfare. Additionally, this framework provides insights into how different policies might influence housing choices and the well-being of retirees.

To sum up, our modeling framework stands out for the following reasons: Firstly, it is very general, incorporating various scenarios such as *SU* with and without housing, and *RU* without housing, allowing for comprehensive comparisons across these settings. Secondly, our framework strikes a balance between realism and simplicity, which enables us to derive analytical solutions in some scenarios. Thirdly, we utilize an off-the-shelf model with widely recognized parameters, ensuring that our results are consistent across different parameter values. Lastly, our study makes a significant contribution to existing literature by quantifying welfare losses in multiple suboptimal retiree scenarios, such as non-participation in stock and bond markets, and improper investment levels in these markets, as well as over or under-consumption of housing and other goods. To our knowledge, no previous research has explored all these suboptimal conditions comprehensively.

## 4 The model

According to the U.S. Census Bureau, in 2050, about 21% of the U.S. population will turn 65 or older. Since society is aging and we do not aim to explain whole life-cycle allocations, we model only the retirement horizon similar to several other studies [34–36, 48–51] in the related literature. We consider a partial equilibrium economy populated by a retiree facing risk about the stock price.

### 4.1 Preferences

We assume that retirees have Epstein-Zin type *RU*. Recursive Utility is about how people think about their satisfaction not just based on what’s happening right now, but also on how they expect to feel in the future. It’s like a chain reaction, where each decision about satisfaction is linked to the next.

The retiree has an uncertain lifetime, but is definitely dead after time *T*. The utility of a representative retiree with an uncertain lifetime is given as follows:

$$\begin{array}{c} U_{t}=f\left(cc_{t},\mu_{t}\left(U_{t+1}\right)\right)\;\text{for}\;0\leq t<T\;\text{and}\;U_{T}=C_{T}^{1-\psi }H^{\psi } \end{array}$$ (1)

$$\begin{array}{c} cc_{t}=C_{t}^{1-\psi }H^{\psi } \end{array}$$ (2)

Periodic utility (*U*_*t*_) is a function of *cc*_*t*_ and *μ*_*t*_(*U*_*t*+1_). The composite consumption (*cc*_*t*_) denotes the composite of non-housing consumption (*C*_*t*_) and housing (*H*) at time *t*. Utility in the last period (*T*) is denoted by *U*_*T*_ and is the Cobb-Douglas aggregator of time *T* non-housing consumption (*C*_*T*_) and the quantity of housing (*H*). The Cobb-Douglas aggregator is like a recipe that economists use to mix different ingredients, like consumption and housing, to understand how they come together to produce composite consumption. The parameter *ψ* stands for the weight of housing in the utility.

The function *f* is an aggregator and *μ*_*t*_(*U*_*t*+1_) denotes the certainty equivalent of the distribution of time *t* + 1 utility, *U*_*t*+1_, conditional upon information available at time *t*. Certainty equivalent of the distribution of time t + 1 utility, conditional upon information available at time t is the best guess about tomorrow’s happiness based on the information possessed right now.

The aggregator *f* is given below:

$$\begin{array}{c} f\left(cc_{t},v_{t}\right)={\left[cc_{t}^{\phi }+\beta \rho_{t}v_{t}^{\phi }\right]}^{1/\phi };\;\phi <1,\;\beta >0 \end{array}$$ (3)

where *β* > 0 denotes the time discount factor. Time discount factor is a measure of how much less we value things the further away they are in time. So, if the time discount factor is low, it means we really prefer things now rather than later. If it’s high, it means we’re more patient and don’t mind waiting for things.

The parameter *ϕ* symbolizes the parameter of the elasticity of intertemporal substitution(*EIS*). *EIS* is a fancy way of talking about how willing people are to change when they spend or save their money over time. People who are very flexible and happy to switch their spending from today to tomorrow have high elasticity. *EIS* equals to $\frac{1}{1-\phi }$. The certainty equivalent of the future utility(*μ*_*t*_) is like a guaranteed level of happiness or satifaction you’d accept now instead of taking a chance on possibly being happier in the future.

$$\begin{array}{c} \mu_{t}={\left(E_{t}U_{t+1}^{\alpha }\right)}^{1/\alpha }; \end{array}$$ (4)

where *E*_*t*_ denotes the expected value conditional upon information available at time *t*.

The parameter *ρ*_*t*_ stands for the conditional survival probability. In other words, *ρ*_*t*_ is the probability that the retiree will be alive at time *t* + 1 given that he is alive at time *t*. The parameter *α* denotes the parameter of risk aversion. More precisely, 1 − *α* is the degree of relative risk aversion (*RRA*). *RRA* is like a scale that shows how much of a thrill-seeker people are when it comes to their finances. If people have high degrees of risk aversion, they prefer safer investments, even if those offer lower returns. If people have low degrees of risk aversion, they are willing to take on more risk for the chance of higher returns.

When *α* = *ϕ*, we obtain the power specification of the *SU* where both of *RRA* and *EIS* are controlled by the single parameter *α*. Accordingly, *EIS* has to be small if *RRA* is large. To independently adjust *RRA* and *EIS*, allowing for scenarios where both can be either high or low simultaneously, different parameters should be used for each. This separation allows for more flexibility in modeling preferences regarding risk and time.

We obtain recursive utility, *U*_*t*_, for time *t* ∈ {0, 1, 2, …, *T* − 1} by combining the aggregator and certainty equivalent.

$$\begin{array}{c} U_{t}={\left[{\left(C_{t}^{1-\psi }H^{\psi }\right)}^{\phi }+\beta {\left(\rho_{t}E_{t}U_{t+1}^{\alpha }\right)}^{\phi /\alpha }\right]}^{1/\phi }; \end{array}$$ (5)

### 4.2 Wealth and budget constraint

The individual makes the following decisions at *t* ≥ 1: how much of wealth to consume (*C*_*t*_), how much of wealth to invest in the risk-free bond (*B*_*t*_), and how many risky stocks to buy (Π_*t*_) given the initial endowment (*W*_0_) on a binomial tree (In finance, binomial tree helps us understand how things like stock prices can change over time, with each step having two possible outcomes: up or down.). Hence, the retiree is subject to the following budget constraint:

$$\begin{array}{c} B_{t}+C_{t}+\Pi_{t}S_{t}=W_{t} \end{array}$$ (6)

The person allocates his liquid wealth (*W*_*t*_) between consumption (*C*_*t*_), bond investment (*B*_*t*_) and stock investment (Π_*t*_*S*_*t*_).

$$\begin{array}{c} W_{t}=B_{t-1}R+\Pi_{t-1}S_{t} \end{array}$$ (7)

where *W*_*t*_ is the sum of time *t* returns from time *t* − 1 investment in bonds and in stocks. *R* denotes the gross risk-free interest rate where *R* = 1 + *r*. As in [52, 53], we abstract from labor income or retirement benefits.

The retiree’s budget constraint at *t* = 0 is given below:

$$\begin{array}{c} B_{0}+C_{0}+\Pi_{0}S_{0}+P_{0}^{H}H=W_{0} \end{array}$$ (8)

We assume that the person retires at *t* = 0 and buys a house to live in until he dies. Hence, at *t* = 0, in addition to deciding *C*_0_, *B*_0_ and *S*_0_, the person also decides the quantity of housing (*H*) given the initial price of housing ($P_{0}^{H}$) and the initial stock price (*S*_0_).

We assume that the retiree is a homeowner during his entire retirement period because most of the elderly own their houses free of debt according to the micro data of retirees in the *SCF*. Accordingly, our model does not include a rental market, similar to the approach by [54]. While some academics might view this realistic assumption as a limitation, our focus is not on why retirees choose to own rather than rent. Instead, we are interested in exploring the costs associated with suboptimal retiree decision of living in a house larger than his/her need and some other deviations in the housing decision. Therefore, including a rental market does not align with our primary objective, making it irrelevant to our analysis.

Our model assumes that retirees purchase a home upon retirement and maintain ownership until death, reflecting data from the *SCF* which indicates that elderly individuals are unlikely to move unless faced with a catastrophic event. This assumption simplifies our model by limiting the retiree to a single housing decision during retirement. Since this approach is supported by the micro data of the elderly, it helps keep our model realistic without unnecessarily complicating it.

It might be questioned whether our model’s assumption—that retirees do not change their homes—is a limitation. However, our goal isn’t to explore why retirees remain in the same house. Instead, we are focused on assessing the welfare costs associated with retirees living in homes that deviate from their optimal housing needs. Given this specific aim, the decision to potentially change houses during retirement is not relevant to our analysis. This approach keeps our model focused and directly aligned with our research objectives.

[55] analyze the Consumer Expenditure Survey(*CEX*) and find that the market value of housing services peaks at age 55, then slightly decreases, and stabilizes for the remainder of the life cycle. [56] utilizes *SCF* data to study housing stock per adult-equivalent throughout the life cycle, discovering that housing stock grows until age 65 before leveling off. While [57] examines how an increase in non-market time due to retirement contributes to the observed housing patterns among retirees [58], investigates how habit formation affects retirees’ housing choices. These studies collectively shed light on the complex factors influencing the housing decisions of retirees.

In our model, we treat housing strictly as a consumption good rather than an investment, limiting retirees to a single housing decision. This approach aligns with observations from microdata on the elderly and studies by researchers like [24], which suggest that the elderly typically view housing primarily as a permanent residence rather than an investment asset. This perception supports our modeling choice and sheds light on asset allocation decisions among the elderly. By keeping the housing decision constant, our model remains realistic and allows us to derive analytical solutions effectively.

In our model, we use the same rates for both lending and borrowing, and we do not consider labor income shocks or transaction costs in house trading. This simplification, which is also common in many studies about the elderly, helps make the model more manageable. Although including transaction costs could potentially strengthen our findings, their exclusion does not prevent us from drawing clear conclusions about the economic behaviors of retirees. Thus, our approach remains effective for analyzing the financial decisions of the elderly without these additional complexities.

A pension is the income a person sets aside while working so they will be able to get a monthly paycheck after retirement. Virtually all pension arrangements allow to take a tax-free lump sum at retirement. Since many retirees feel they are likely to earn a higher return by investing a lump sum than they—d get with a monthly income, they choose the lump sum. Managing a lump sum allows a person to directly address risks such as inflation, investment volatility, and longevity by adapting investment strategies over time. This might involve shifting asset allocations or changing spending patterns as necessary.

In our model, the initial wealth (*W*_0_) can be considered as the sum of all the savings of the agent before retirement and the pension payout which can be thought of as either a lump sum or the present value of future monthly payments. According to consulting firm Willis Towers Watson, about 50% to 80% of workers choose the lump sum, depending on factors including the company and industry. Hence, our modelling of initial wealth is not unrealistic. Several studies [32, 34–36, 59] use initial wealth instead of monthly pension income. Thus, our way of modelling is common. In addition, assuming that the borrowing and lending rates are the same, a lump sum payment for a retiree can have a similar impact on the budget constraint with another retiree who borrows this amount and pays it with their monthly fixed income.

While modeling the pension income as a lump sum helps us to obtain closed-form results, the intuition for our results is general. Initial wealth represents liquid assets that individuals have direct control over, allowing them to make immediate investment decisions. By focusing on initial wealth, the model can concentrate on the decision-making process related to liquid assets. By excluding pension income, the model can provide a clearer picture of the risks associated with different investment choices and the impact on the sustainability of retirement funds. Excluding pension income can make the model more universally applicable, as pension schemes vary widely across different countries and sectors. By focusing on initial wealth, the model can be more easily compared and adapted to different contexts.

### 4.3 Financial assets

The retiree invests in the risk-free bond with gross return *R* and in the risky stock. At time *t* ≥ 1, the two possible states for the stock price are ($S_{t}^{u}$, $S_{t}^{d}$) such that

$$\begin{array}{c} S_{t}=S_{t}^{u}\;\text{with}\;\text{probability}\;\text{p}\;\text{and}\;S_{t}=S_{t}^{d}\;\text{with}\;\text{probability}\;q=1-p \end{array}$$ (9)

where $S_{t}^{u}=uS_{t-1}$ and $S_{t}^{d}=dS_{t-1}$, 0 < *d* < 1 < *u* given the initial stock price. In the upward state, stock prices increase by a factor of *u* compared to their previous value, while in the downward state, they decrease by a factor of *d*. Stock prices have a chance of increasing, which is expressed as *p*, and a chance of decreasing, denoted as *q* = 1 − *p*. We assume that the interest rate is constant and deterministic similar to [33, 60].

### 4.4 Parametrization

Time is discrete. A household retires and enters our economy at age 65 (*t* = 0). Given the significant uncertainty retirees in the U.S. face regarding lifespan, our model assumes that the retiree could pass away at any time but will not live beyond age 80.

We have an annual decision frequency. We use the conditional survival rates provided by [61]. We set the risk-free interest rate (*r*) at 2% which is a commonly used value for *r* in the related literature (e.g. [33]). We assume that the real stock return has a mean of 6% and standard deviation of 18% aligning with the values used in several papers (e.g. [33, 62]).

The weight of housing services in the composite consumption good, *ψ*, measures how much the person values housing relative to the consumption of other goods. We set *ψ* at 0.2 similar to several studies (e.g. [33, 60]) in the housing literature. Regarding risk aversion (*RRA*) [63], present empirical evidence and propose a range between 2 and 5. Thus, the exact value of *RRA* is a matter of taste as long as it is from the proposed range. We set *α* at -2 to correspond to an *RRA* value of 3, fitting within the accepted empirical range.

In the literature, *ϵ* = 1/(1 − *ϕ*) is used as a parameter of *EIS*. The estimated value for *ϵ* is 0.1 in [64], 0.4 in [65] and 1 in [66]. It stays in a range from 0.05 to 1, with clusters around 0.25 and 0.7 in [67]. We also set *ϵ* at 0.7. Furthermore [68, 69], suggest a value of 0.3 using aggregate data and 0.8 using cohort data.

We use a Cobb-Douglas utility function to combine housing services and non-housing consumption, a common approach in the housing literature, as seen in studies such as [60, 33]. Finally, we choose the subjective time discount rate parameter (*β*) to match the model generated stock over liquid savings ratio with its value in the data ([33] calculated the average stock proportion in liquid financial assets as 41% for the people aged 65–75. When *β* = 0.955, our calibrated value for the average stock proportion becomes 42% which is very close to the corresponding estimated value.). The resulting value of *β* lines up with its standard estimates. We calibrate our model to the data with the determination of *β*. However, as shown in Section 7, the calculated welfare costs are robust and do not vary significantly with different values of *β*, confirming the stability of our model across different scenarios.

We assign the parameters to the commonly used values in the housing literature, mainly adopting the settings from [33], except for the parameter *ϵ*. Since [33] do not include recursive utility (*RU*) and thus do not use *ϵ*, we adopt this parameter’s value from the influential study by [67], which is commonly referenced in discussions of *RU*. This approach ensures that our model is consistent and credible, drawing from recognized sources within the field.

### 4.5 Analytical solution of optimal retiree decisions

The analytical solution of the optimal retiree allocations is presented below.

**Definition 1** *For t* ≥ 0

$$\begin{array}{ccc} B_{t} & = & b_{t}W_{t}\\ C_{t} & = & c_{t}W_{t}\\ \Pi_{t}S_{t} & = & \pi_{t}W_{t} \end{array}$$ (10)

*where B_t_ denotes the dollars invested in bond*, Π_*t*_*S*_*t*_ *denotes dollars invested in stock, C_t_ represents the dollars consumed*, Π_*t*_ *denotes the number of stock holdings, W_t_ stands for the liquid wealth and S_t_ denotes the stock price at time t*.

*The variable c_t_ denotes the fraction of time t liquid wealth* (*W*_*t*_) *used for time t consumption. The variables* (*b*_*t*_, *π*_*t*_) *denote the fraction of the dollars invested in bonds and stocks as a percentage of liquid wealth*.

Starting from the last period *T*, we find (*b*_*t*_, *c*_*t*_, *π*_*t*_) by using the technique of backward iteration, and then (*B*_*t*_, *C*_*t*_, Π_*t*_) by going forward in time.

**Proposition 1**. *The retiree decisions at time t* > 0 *are as below: where*

$$\begin{array}{c} b_{t}=\frac{l}{1+l+m_{t}\left(d+Rl\right)} \end{array}$$ (11)

$$\begin{array}{c} c_{t}=\frac{m_{t}\left(d+Rl\right)}{1+l+m_{t}\left(d+Rl\right)} \end{array}$$ (12)

$$\begin{array}{c} \pi_{t}=\frac{1}{1+l+m_{t}\left(d+Rl\right)} \end{array}$$ (13)

*where*

$$\begin{array}{ccc} k & = & {\left[\frac{(d-R)q}{p(R-u)}\right]}^{\frac{1}{(1-\psi )\alpha -1}}\\ l & = & \frac{kd-u}{R(1-k)} \end{array}$$

$$\begin{array}{c} g_{t}=p{\left(b_{t}R+u\pi_{t}\right)}^{(1-\psi )\alpha }\\ z_{t}=q{\left(b_{t}R+d\pi_{t}\right)}^{(1-\psi )\alpha }\\ u_{t}={\left(c_{t}^{(1-\psi )\phi }+\beta {\left[g_{t}+z_{t}\right]}^{\phi /\alpha }\right)}^{1/\phi } \end{array}$$

$$\begin{array}{c} \beta_{t}=\beta \rho_{t}u_{t+1}^{\phi } \end{array}$$

$$\begin{array}{c} m_{t}={\left\{\beta_{t}{\left[pk^{(1-\psi )\alpha }+q\right]}^{\frac{\phi -\alpha }{\alpha }}\left(puk^{(1-\psi )\alpha -1}+qd\right)\right\}}^{\frac{1}{(1-\psi )\phi -1}} \end{array}$$

In Proposition 1, we show that the decisions about consumption, bonds, and stocks in each period (represented by *c*_*t*_, *b*_*t*_, *π*_*t*_ respectively) are independent of the initial amount of money someone starts with, denoted as *W*_0_.

We introduce the variables (*k*, *l*, *g*_*t*_, *z*_*t*_, *u*_*t*_, *β*_*t*_, *m*_*t*_) for two main reasons. First, using these variables makes the formulas for determining how much to consume and invest in stocks and bonds simpler and more straightforward. Second, these variables help us to solve the problem recursively, step-by-step in a way that builds on previous steps, which makes finding the solution faster. Explanations of these equations can be found in S1 Appendix.

## 5 Simulation analysis

First, we simulate stock returns and then use our optimal decision rules from the analytical solution. The age profiles of the optimal decisions are generated by repeating the calculation from *t* = 0 (age 65) to *t* = 15 (age 80) for 50,000 simulation paths.

**Fig 1:**
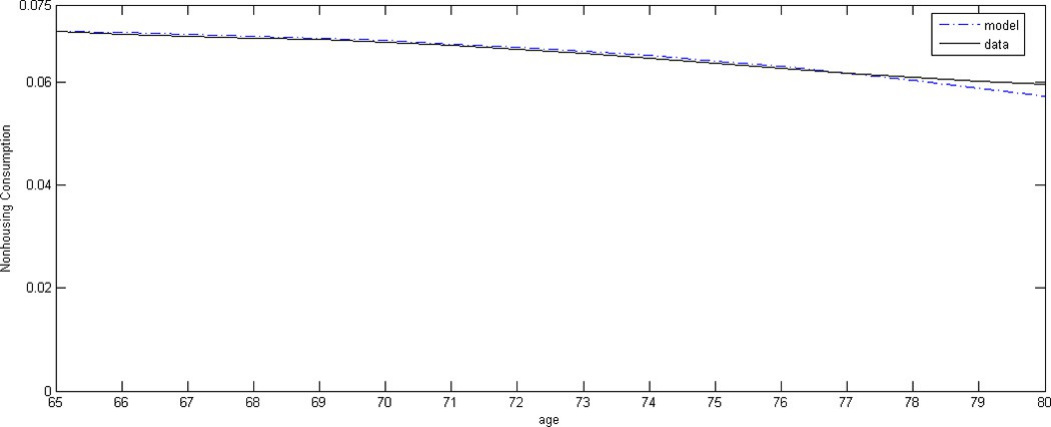
Non-housing consumption.

Fig 1 shows the consumption profile of the model and the data from *SCF*. As seen in the figure, our calibrated model mimics the consumption data for homeowners well. Since we assume that the agent buys a house initially and uses it thereafter, we compare our model result with the consumption data of homeowners.

## 6 Suboptimal allocations

As we stated before, when retirees don’t use their money and assets in the best way possible (suboptimal decision making), they might not get as much happiness or satisfaction (utility) as they could. Even if they think they’re making smart choices, they might not be making the best ones (optimal) because there are other things at play besides just risk and return.

People often make financial choices based on their feelings (psychological biases) and common shortcuts (heuristics) in thinking. For instance, they might prefer to avoid losses (loss aversion) rather than make gains, stick with what they know (status quo bias), or think they know more than they do (overconfidence). These habits can cause retirees to make choices that aren’t the best for them. But our focus isn’t on why they make these choices; we’re interested in how much these choices could cost them in terms of welfare.

Even when a person can find his/her choices that stem from an optimization process, he can still make part of the decisions suboptimally. For example, let’s say someone is too nervous about the risks of the stock market and keeps more of their money in bonds than might be wise, but they’re spending just fine, and they still have enough money to cover everything. This person might be over-careful because they really don’t want to lose money, and they feel safer with bonds. They don’t change how much they save overall, but how they split their money between stocks and bonds could be better.

As another example, if there’s a strong shopping culture (consumerism), people might be tempted to spend more now instead of saving for later. Suppose someone spends more than they ideally should and saves the right fraction of savings in bonds. In this case, they’re spending too much now, which might hurt their financial security in the future. The issue here isn’t how they’re saving but how much they’re saving, which can be influenced by wanting to buy things now rather than save for later. While the composition of savings (between bonds and stocks) remains optimal, the suboptimality is in the level of savings.

If the retiree decides to have one of the allocations at a suboptimal level and decides the rest of the allocations optimally, as explained in the examples above, he causes a certain degree of inefficiency. We use two different measures to quantify the inefficiency of a suboptimal allocation *x*.

We define $\bar{W_{0}}\left(EU,x\right)$ as the amount of initial wealth necessary to achieve a specific level of expected utility (*EU*) under the allocation *x*. Suppose x* is the investor’s optimal allocation. $\bar{W_{0}}\left(EU\left(W_{0},x^{*}\right),x\right)$ is the necessary initial wealth, under allocation *x*, to attain the same level of expected utility as is achieved with the optimal allocation and initial wealth *W*_0_. Since x* is the optimal allocation, it follows that $\bar{W_{0}}\left(EU\left(W_{0},x^{*}\right),x\right)>W_{0}$. This implies that a higher initial wealth is required when adopting a suboptimal strategy in order to reach the level of utility obtained with the optimal allocation.

We define Ω(*x*) as the percentage increase in initial wealth required under a suboptimal allocation, *x*, to the wealth necessary under the optimal allocation relative to the wealth needed under the optimal strategy, x*, to achieve the same level of utility. Mathematically, Ω(*x*) is expressed as $(\bar{W_{0}}/W_{0}-1)*100$ where *W*_0_ is the initial wealth needed under the optimal strategy, x*. This measure, Ω(*x*), quantifies the inefficiency introduced by choosing a suboptimal allocation. For instance, if Ω(*x*) is five percent, it means that the retiree must have five percent more initial wealth under the suboptimal allocation *x* compared to the optimal allocation, x*, to attain the same level of utility.

We introduce the concept of utility cost as a second metric to assess welfare. The utility cost is represented by *UC*(*x*) where $UC\left(x\right)=\left(U_{0}^{S}/U_{0}^{*}-1\right)*100$. Here, $U_{0}^{S}$ is the utility at the start of retirement under the suboptimal allocation *x* and $U_{0}^{*}$ is the utility when decisions are made optimally. This metric, *UC*(*x*) quantifies the reduction in utility due to suboptimal decision-making. We analyze both Ω(*x*) and *UC*(*x*) across various suboptimal allocations to understand the implications on retiree welfare.

### 6.1 Suboptimal stock holdings

#### 6.1.1 The analytical solution for zero stock holding case

If the agent chooses not to allocate any funds into stocks, the optimization problem is redefined as follows:

$$\begin{array}{c} \text{max}{\left[{\left(C_{t}^{1-\psi }H^{\psi }\right)}^{\phi }+\beta \rho_{t}{\left(E_{t}U_{t+1}^{\alpha }\right)}^{\phi /\alpha }\right]}^{1/\phi } \end{array}$$

subject to:

*C*_*t*_ + *B*_*t*_ = *W*_*t*_ and *W*_*t*_ = *B*_*t*−1_*R* for *t* ≥ 1 and $C_{0}+P_{0}^{H}H_{0}+B_{0}=W_{0}$

The person splits his time *t* (*t* > 0) liquid wealth, *W*_*t*_, between consumption (*C*_*t*_) and bond investments (*B*_*t*_). This liquid wealth (*W*_*t*_) is derived from the accumulated savings in bonds from the previous period. At *t* = 0, the person divides his/her wealth among buying a house ($P_{0}^{H}H_{0}$), consumption (*C*_0_), and bonds (*B*_0_).

We introduce a novel analytical recursive solution to this problem, which we outline in the following steps:

**Step 1**: Set *t* = *T*, *β*_*T*_ = *β*, *c*_*T*_ = 1, *u*_*T*_ = 1, and *b*_*T*_ = 0.
**Step 2**: Set *t* = *T* − 1
**Step 3**: Find *m*_*t*_, *c*_*t*_, *u*_*t*_, and *b*_*t*_ where
  $$\begin{array}{c} c_{t}=\frac{m_{t}}{1+m_{t}}\;\text{and}\;m_{t}={\left(\beta_{t}\right)}^{\frac{1}{(1-\psi )\phi -1}}\;\text{and}\;\beta_{t}=\beta \rho_{t}R^{(1-\psi )\phi }u_{t+1}^{\phi }\\ b_{t}=\frac{1}{1+m_{t}}\;\text{and}\;u_{t}=\{c_{t}^{(1-\psi )\phi }+\beta_{t}b_{t}^{(1-\psi )\phi }{\}}^{1/\phi } \end{array}$$
**Step 4**: Set *t* = *t* − 1. If *t* > 0, go to Step 3. Otherwise, go to Step 5.
**Step 5**: Solution at time *t* = 0 is given below:
  $$\begin{array}{ccc} \beta_{0} & = & \beta \rho_{0}R^{(1-\psi )\phi }u_{1}^{\phi }\\ m_{0} & = & {\left(\beta_{0}\right)}^{\frac{1}{(1-\psi )\phi -1}}\\ n_{0} & = & \frac{\psi \left[m_{0}^{(1-\psi )\phi }+\beta_{0}\right]}{\left(1-\psi \right)m_{0}^{(1-\psi )\phi -1}}\\ c_{0} & = & \frac{m_{0}}{1+m_{0}+n_{0}}\\ b_{0} & = & \frac{1}{1+m_{0}+n_{0}}\\ h_{0} & = & \frac{n_{0}}{1+m_{0}+n_{0}}\\ u_{0} & = & \{c_{0}^{(1-\psi )\phi }+\beta_{0}b_{0}^{(1-\psi )\phi }{\}}^{1/\phi } \end{array}$$
**Step 6**: Find *B*_0_ and *C*_0_ where *B*_0_ = *b*_0_*W*_0_, *C*_0_ = *c*_0_*W*_0_, given *W*_0_.
**Step 7**: Set *t* = 1
**Step 8**: Calculate the liquid wealth (*W*_*t*_) using the equation *W*_*t*_ = *B*_*t*−1_*R* given the gross interest rate (*R*).

Calculate bond holdings (*B*_*t*_) and consumption (*C*_*t*_) such that *B*_*t*_ = *b*_*t*_*W*_*t*_ and *C*_*t*_ = *c*_*t*_*W*_*t*_.

**Step 9**: Set *t* = *t* + 1. If *t* ≤ *T* then go to Step 8. Otherwise, stop.

We obtain a utility cost of 3.71% for retirees who choose not to invest in stocks. We also find that a retiree would need an additional 3.86% in initial wealth to achieve the same level of utility that they would have under an optimal investment scenario that includes stocks. Thus, we conclude that opting out of stock market participation does not result in a significant financial disadvantage for retirees. This suggests that while investing in stocks can enhance financial well-being, the absence of such investments isn’t drastically detrimental.

[70] suggest that entering the stock market involves costs such as learning about the market’s operations. Other potential costs include monitoring investments, making decisions, paying brokerage and transaction fees, and spending extra time on tax filings. In our analysis, we exclude these costs to simplify our model. However, it’s important to note that by not participating in the stock market, a retiree avoids these fees entirely. Therefore, when these costs are considered, the welfare loss from not investing in stocks is even less significant. This actually reinforces our finding that the welfare cost of not investing in stocks is low. This suggests that the impact of missing out on stock market gains may be somewhat offset by the savings on these associated costs.

We simplify our model by not including the complexities of decision-making, but it’s worth noting that if we considered these complexities, the welfare losses from not participating in the stock or bond markets might actually be lower. This is because by avoiding these markets, a retiree also avoids the complexities and burdens of financial planning associated with them. Therefore, not participating in the stock market may not be as detrimental as it seems, given the low welfare cost and reduced decision-making complexity. This suggests that the decision to stay out of the stock market doesn’t significantly harm financial well-being.

#### 6.1.2 Positive suboptimal stock holdings

Suppose the retiree decides to invest a fraction of the optimal amount in stock (as a fraction of his current wealth) during his entire retirement and then makes consumption, housing and bond investment decisions optimally. We solve the model numerically in this case because of nonlinearity problems.

**Fig 2:**
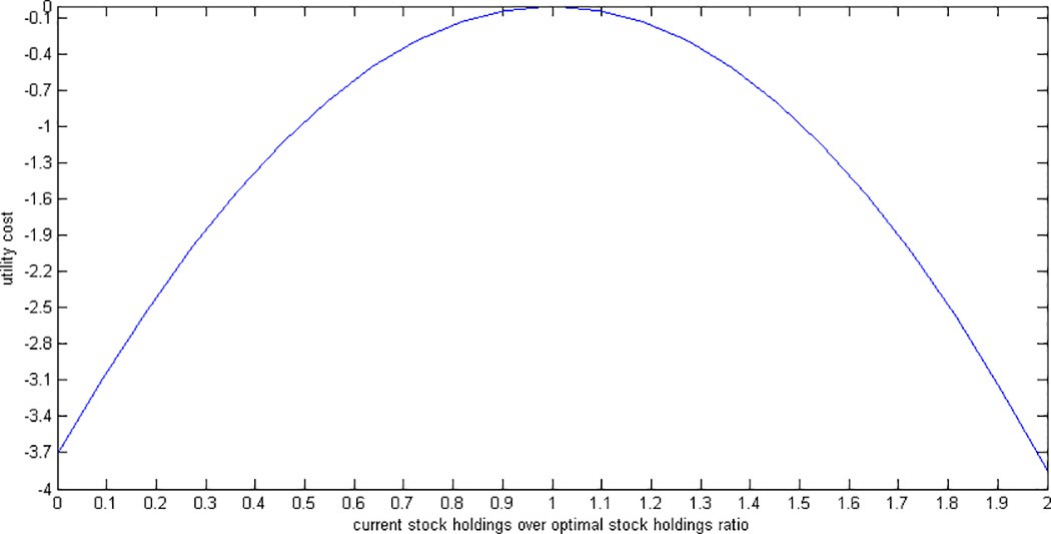
Utility cost of suboptimal stock holdings.

Fig 2 shows the utility costs of various deviations in a stock investment decision from the optimal level. It shows that if the agent invests 50% of the optimal level in stocks, the utility cost is around -1%. In other words, the utility at the start of the retirement under the suboptimal scenario is 1%less than that under the optimal scenario.

**Fig 3:**
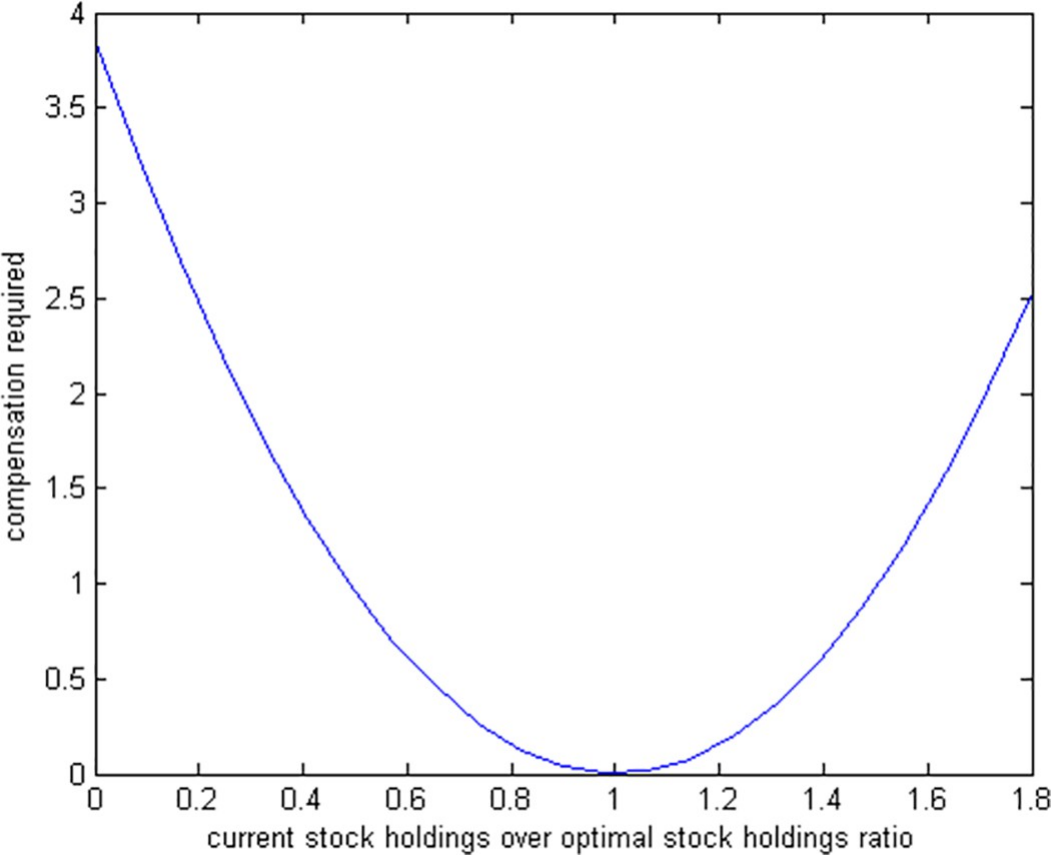
Compensation required for suboptimal stock holdings.

Fig 3 shows that the necessary compensation is around 0.8% if the agent holds 50% of the optimal stock holdings. It implies that the agent needs only 0.8% more initial wealth to reach the level of optimal utility.

We find that the utility cost of making suboptimal investment decisions—either over-investing or under-investing in stocks by up to 100%—remains below 3.8% for over-investments and 3.7% for under-investments. Similarly, the compensation needed to offset these suboptimal decisions is less than 4% for over-investments and 3.8% for under-investments, with over-investment generally resulting in slightly higher costs than under-investment. If we factor in the additional fees associated with stock investments, such as brokerage and transaction fees, the cost of over-investment would likely increase more than that of under-investment. Therefore, incorporating these investment fees would further support our findings that over-investment is more detrimental than under-investment.

Studies, like those by [22], highlight that even wealthy individuals often invest little in stocks. Our analysis supports this observation, showing that under-investment in stocks does not lead to significant welfare losses for households. Therefore, the low level of stock investment among retirees may not be a critical issue in terms of improving their welfare. This suggests that focusing on increasing stock investment levels might not be necessary for enhancing the financial well-being of retirees.

The utility costs in *SU* are around twice of those in *RU*. Thus, using *RU* decreases the utility costs of suboptimal stock holdings. On the other hand, utility costs are low under both settings.

As seen in figures, the utility costs associated with different deviations in a stock investment decision from the optimal level are negative. As mentioned before, the best (optimal) choices are those that make a person the happiest (maximum utility). A negative utility cost makes sense as when someone does not make the best choices, they end up being less happy than they could be if they had made the best possible (optimal) choices. Hence, the economic intuition behind a negative cost is that not choosing wisely can cost you some happiness.

### 6.2 Suboptimal bond holdings

#### 6.2.1 Zero bond holdings

If the investor chooses not to invest in the risk-free bond, the optimization problem is reconfigured as follows:

$$\begin{array}{c} \text{max}{\left[{\left(C_{t}^{1-\psi }H^{\psi }\right)}^{\phi }+\beta \rho_{t}{\left(E_{t}U_{t+1}^{\alpha }\right)}^{\phi /\alpha }\right]}^{1/\phi } \end{array}$$

subject to:

*C*_*t*_ + Π_*t*_*S*_*t*_ = *W*_*t*_, and *W*_*t*_ = Π_*t*−1_*S*_*t*_ for *t* ≥ 1 and $C_{0}+P_{0}^{H}H+\Pi_{0}S_{0}=W_{0}$

The person splits his liquid wealth (*W*_*t*_) between consumption (*C*_*t*_) and stock investment (Π_*t*_*S*_*t*_). This liquid wealth primarily stems from the value of stocks purchased in the previous period. At the beginning of retirement (*t* = 0), the retiree allocates a portion of their initial wealth to purchase a house ($P_{0}^{H}H$). We also introduce a recursive solution to this financial decision-making problem, which is a novel contribution of our paper. This solution methodically breaks down the process and is explained below:

**Step 1**: Set *t* = *T*, *β*_*T*_ = *β*, *c*_*T*_ = 1, *u*_*T*_ = 1, and *π*_*T*_ = 0.
**Step 2**: Set *t* = *T* − 1
**Step 3**: Find *m*_*t*_, *c*_*t*_, *u*_*t*_, and *π*_*t*_ using the formulas given below:
  $$\begin{array}{ccc} \beta_{t} & = & \beta \rho_{t}u_{t+1}^{\phi }\{pu^{(1-\psi )\alpha }+qd^{(1-\psi )\alpha }{\}}^{\frac{\phi }{\alpha }}\\ m_{t} & = & {\left(\beta_{t}\right)}^{\frac{1}{(1-\psi )\phi -1}}\\ c_{t} & = & \frac{m_{t}}{1+m_{t}}\\ \pi_{t} & = & \frac{1}{1+m_{t}}\\ u_{t} & = & \left\{c_{t}^{(1-\psi )\phi }+\beta_{t}\pi_{t}^{(1-\psi )\phi }\right\} \end{array}$$
**Step 4**: Set *t* = *t* − 1. If *t* > 0, go to Step 3. Otherwise, go to Step 5.
**Step 5**: We provide the solution for time *t* = 0 below:
  $$\begin{array}{ccc} \beta_{0} & = & \beta \rho_{0}u_{1}^{\phi }\{pu^{(1-\psi )\alpha }+qd^{(1-\psi )\alpha }{\}}^{\frac{\phi }{\alpha }}\\ m_{0} & = & {\left(\beta_{0}\right)}^{\frac{1}{(1-\psi )\phi -1}}\\ n_{0} & = & \frac{\psi \left[m_{0}^{(1-\psi )\phi }+\beta_{0}\right]}{\left(1-\psi \right)m_{0}^{(1-\psi )\phi -1}}\\ c_{0} & = & \frac{m_{0}}{1+m_{0}+n_{0}}\\ \pi_{0} & = & \frac{1}{1+m_{0}+n_{0}}\\ h_{0} & = & \frac{n_{0}}{1+m_{0}+n_{0}}\\ u_{0} & = & \{c_{0}^{(1-\psi )\phi }+\beta_{0}\pi_{0}^{(1-\psi )\phi }{\}}^{1/\phi } \end{array}$$
**Step 6**: Calculate Π_0_ and *C*_0_ such that Π_0_*S*_0_ = *π*_0_*W*_0_ and *C*_0_ = *c*_0_*W*_0_, given the initial endowment (*W*_0_) and the initial stock price (*S*_0_).
**Step 7**: Set *t* = 1
**Step 8**: Find *W*_*t*_ using the equation *W*_*t*_ = Π_*t*−1_*S*_*t*_ and given the gross interest rate (*R*) and the stock price (*S*_*t*_) at time *t*.

Calculate Π_*t*_ and *C*_*t*_ such that Π_*t*_*S*_*t*_ = *π*_*t*_*W*_*t*_ and *C*_*t*_ = *c*_*t*_*W*_*t*_.

**Step 9**: Set *t* = *t* + 1. If *t* ≤ *T* then go to Step 8. Otherwise, stop.

Our analysis finds that not investing in bonds carries a utility cost of about 7%, and a retiree would need approximately 7.5% in compensation. This makes the decision not to invest in bonds roughly twice as costly as not investing in stocks. Therefore, for retirees looking to simplify their investment decisions while minimizing costs, investing in bonds is the advisable choice. This recommendation aligns with observations from the Health and Retirement Study (*HRS*), which shows that many retirees prefer to hold only bonds in their portfolios, corroborating our findings.

#### 6.2.2 Positive suboptimal bond holdings

**Fig 4:**
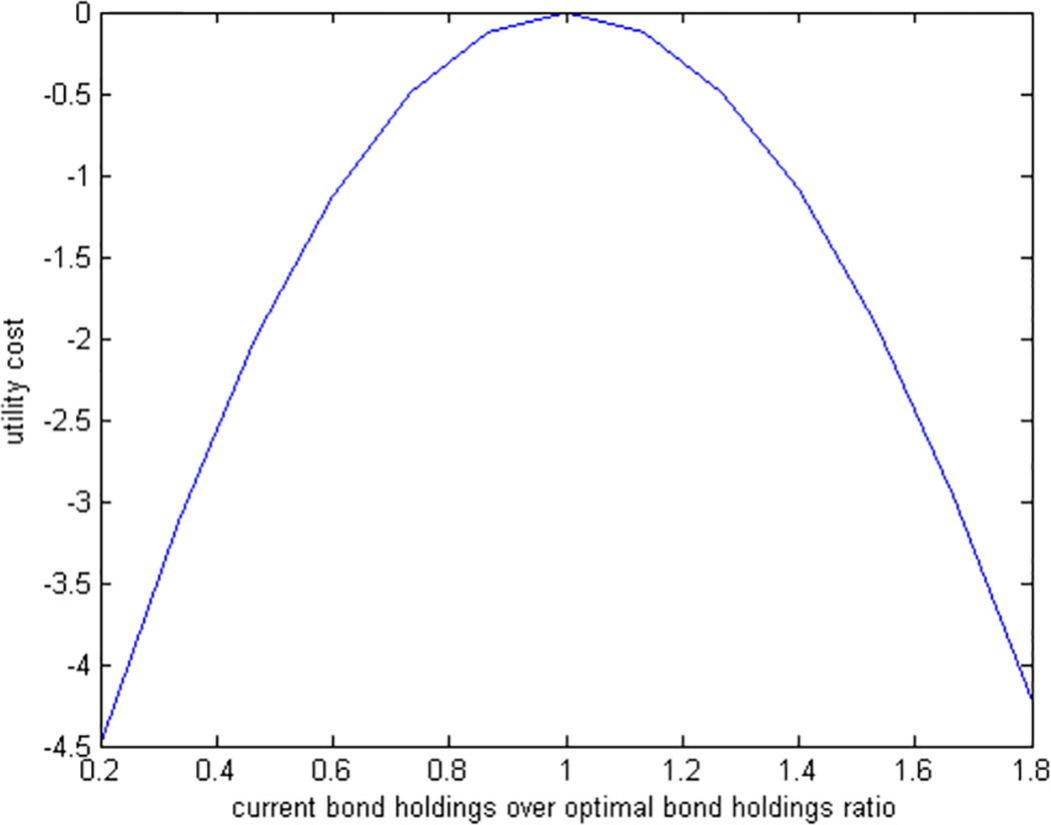
Utility cost of suboptimal bond holdings.

**Fig 5:**
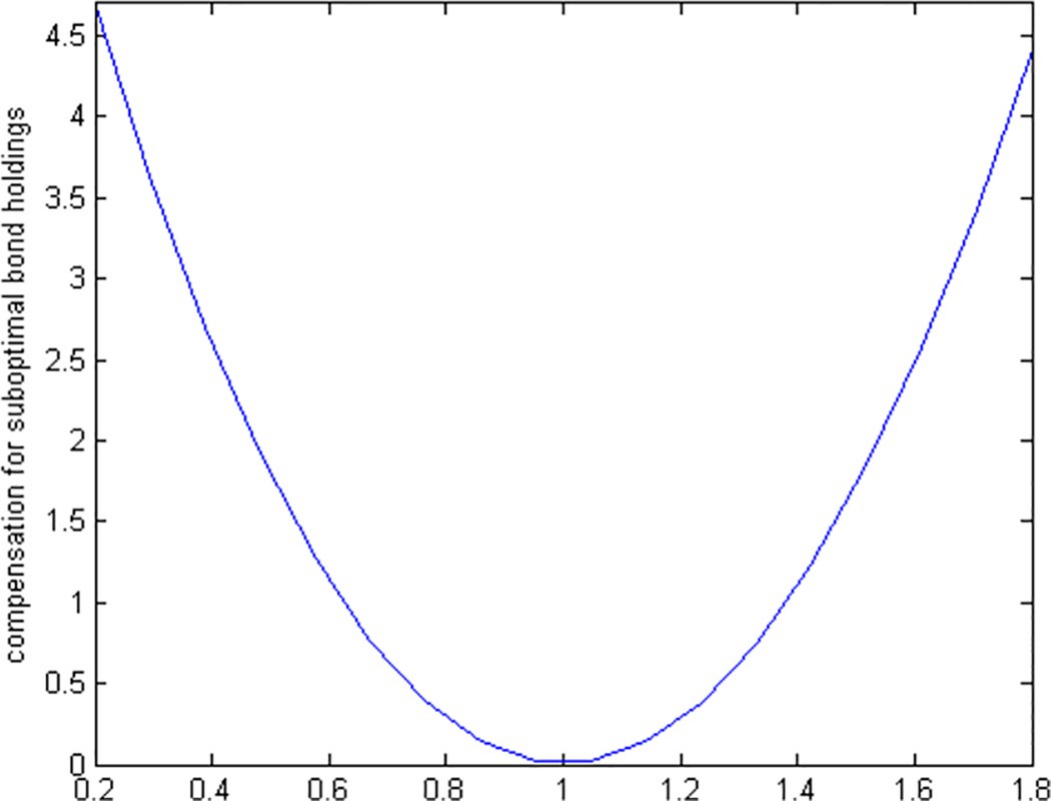
Compensation required for suboptimal bond holdings.

If a retiree chooses to invest a smaller portion of their wealth in bonds than the optimal level suggests, and then makes all other decisions optimally, our model needs to be solved numerically due to the complexity of the nonlinear first-order conditions. Fig 4 displays the utility costs associated with various suboptimal bond investment levels, and Fig 5 details the necessary compensation needed for each level of suboptimal investment. This approach helps us understand the financial impact of not investing the optimal amount in bonds during retirement.

Investing suboptimally in bonds is more costly than doing so in stocks. For instance, the utility loss from investing only 20% of the optimal amount is about 2.4% for stocks but rises to 4.5% for bonds. This suggests that making the right decisions about bond investments is more crucial than for stocks, as errors in bond investment are roughly twice as detrimental. However, even with these differences, the overall impact on welfare from the composition of a liquid portfolio—whether stocks or bonds—is relatively minor. This indicates that the composition of the liquid portfolio does not drastically alter overall financial well-being.

Our analysis shows that making suboptimal bond investments in a *RU* model is roughly twenty times more costly than in a *SU* model. Additionally, the ratio of utility costs between bonds and stocks is higher in *RU*. This suggests that the *RU* model more accurately reflects the importance of bonds relative to stocks in retirees’ portfolios. However, since the overall costs associated with portfolio composition are low in both *RU* and *SU* settings, the exact makeup of a liquid portfolio (how much is invested in stocks versus bonds) does not greatly influence overall financial welfare under either model.

### 6.3 Suboptimal consumption

**Fig 6:**
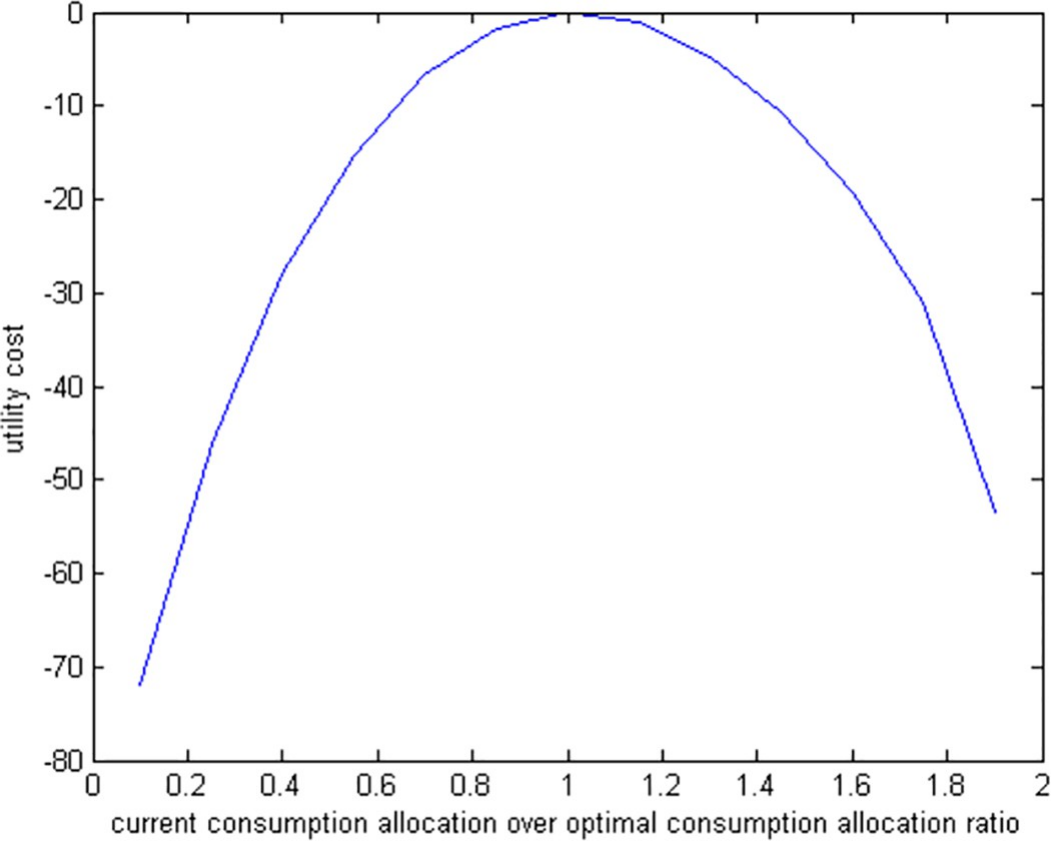
Utility cost of suboptimal consumption allocations.

**Fig 7:**
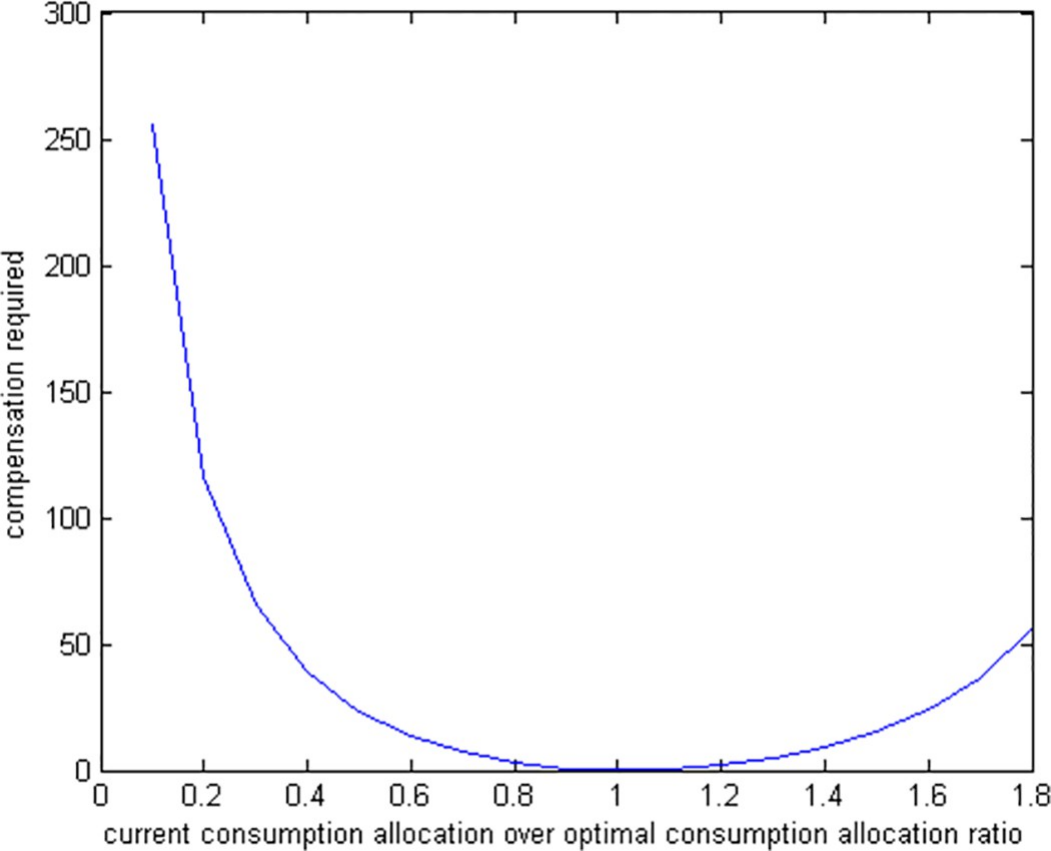
Compensation required for suboptimal consumption allocations.

If a retiree chooses to spend only a fraction of the optimal amount for consumption while making optimal decisions regarding housing, stocks, and bonds, we use numerical methods to solve the model because of its complex nonlinear conditions. To understand the impact of these suboptimal consumption decisions, we run simulations to measure the welfare losses. The results of these simulations are displayed in two figures: Fig 6, which illustrates the utility costs of various suboptimal consumption levels, and Fig 7, which shows the amount of additional wealth needed to compensate for these costs.

We find that the consequences of suboptimal consumption are the most significant compared to other suboptimal financial decisions. For instance, a retiree who spends 50% less than the optimal amount might need a compensation of up to 25% to reach their expected level of well-being. In contrast, a similar underinvestment in bonds only requires a compensation of 1.8%. This highlights that decisions related to how much to consume and save are more crucial to a retiree’s welfare than how their liquid assets are allocated.

We notice that the financial impact of spending too much or too little compared to the optimal amount is not the same. Specifically, a retiree who spends 50% more than the ideal amount requires around 15% additional funds to compensate for the extra spending. In contrast, a retiree who spends 50% less than needed requires about 25% additional compensation. This indicates that spending too little is more detrimental to a retiree’s financial well-being than spending too much to the same extent.

### 6.4 Suboptimal housing

**Fig 8:**
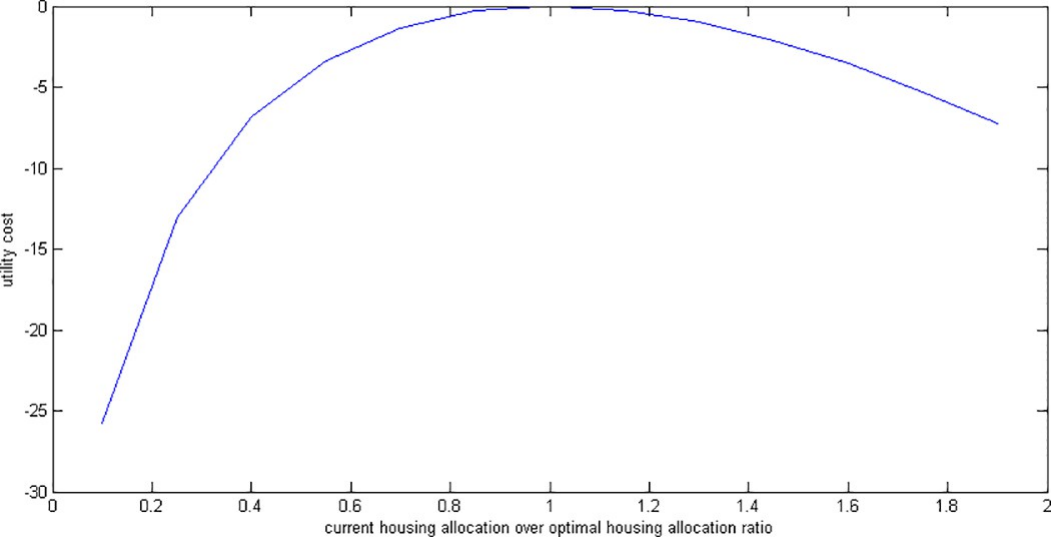
Utility cost of suboptimal housing allocations.

**Fig 9:**
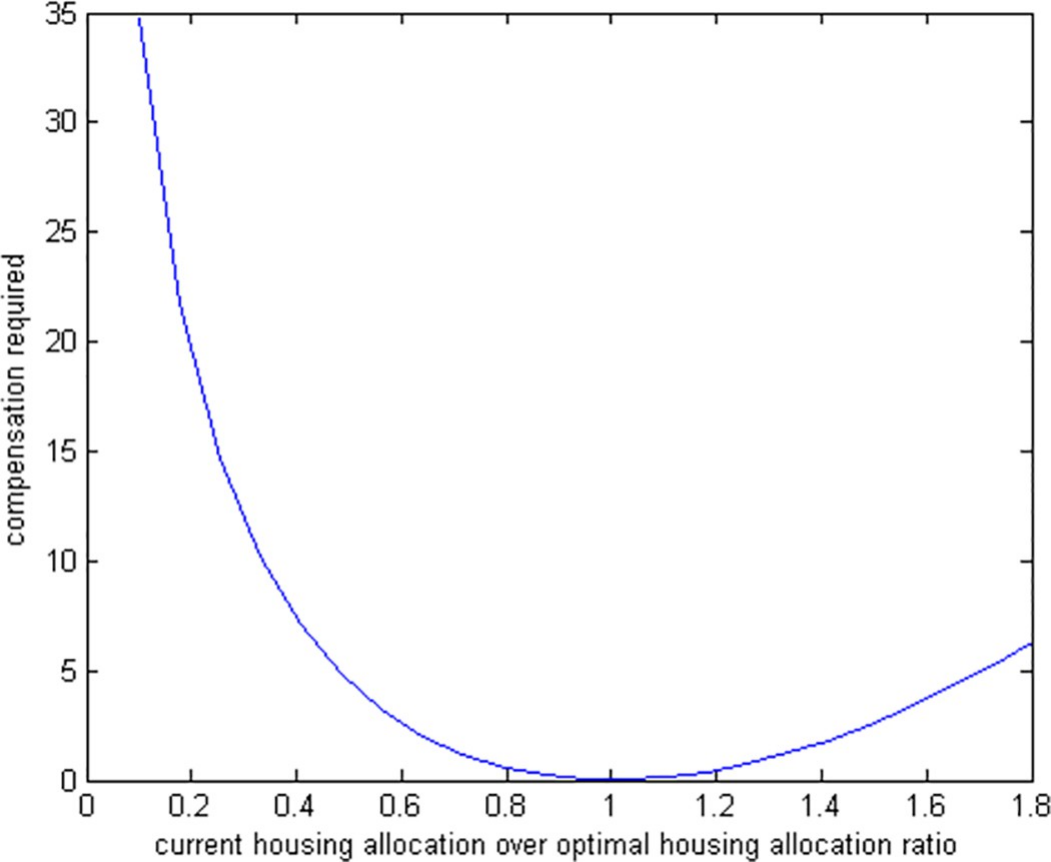
Compensation required for suboptimal housing allocations.

Suppose a retiree decides to allocate a fraction of the optimal amount for housing (as a fraction of current wealth) at the beginning of retirement (*t* = 0) and keeps this house for life. Afterwards, the retiree decides the rest of the allocations optimally. We solve our model analytically. Fig 8 shows utility costs of various suboptimal housing allocations. Fig 9 shows how much extra money would be needed to compensate for these non-optimal housing choices.

**Proposition 2**. *If a retiree decides to hold a fraction of the optimal level of housing; h* = zh*, *such that the budget constraints are not violated, then the optimal retiree decisions at t* = 0 *are given as follows*:

$$\begin{array}{ccc} h & = & zh^{*}\\ \pi_{0} & = & \frac{1-zh^{*}}{m_{0}\left(Rl+d\right)+1+l}\\ b_{0} & = & \frac{l(1-zh^{*})}{m_{0}\left(Rl+d\right)+1+l}\\ c_{0} & = & 1-\frac{\left(1+l\right)\left(1-zh^{*}\right)}{m_{0}\left(Rl+d\right)+1+l}-zh^{*} \end{array}$$

*where z* ≥ 0 and h* *denotes the optimal level of housing as a fraction of wealth. The constants k*, *l*, *m*_0_ *and the household decisions b*_*t*_, *c*_*t*_, *π*_*t*_ *at time t* ⩾ 1 *are defined in Proposition 1*.

**Proposition 3**. *If the retiree buys a house* λ *percent more than the optimal value of housing, then the amounts of relative change in consumption, bond and stock holdings are all the same and equal to* $\frac{\lambda \psi }{1-\psi }$ %.

*Proof*. It follows from the allocations given in Proposition 1.

When a retiree purchases a house that is more expensive (or cheaper) than the optimal value, it’s similar to starting with less (or more) initial wealth while making other decisions optimally. For example, buying a house at 50% above the optimal value results in the same utility as if the retiree had started with 10% less initial wealth but made all other decisions optimally. This illustrates how spending more on housing effectively reduces the available wealth for other uses, impacting overall financial well-being.

The impact of not making the best housing decisions is consistent across different utility functions, regardless of specific parameters like risk aversion, elasticity of intertemporal substitution, or discount rate. However, the costliness of these decisions does vary based on how much weight the individual places on housing in their overall utility. This means that as long as two utility functions assign the same importance to housing, the costs of suboptimal housing decisions will be the same for both functions.

The utility cost of suboptimal housing has the highest degree of asymmetry among all suboptimal cases. The cost of making housing decisions that are not optimal shows a big difference between having too much housing and not enough. For example, if someone has 90% less housing than they should, they need a compensation of 34%. But if they have 90% more housing than they should, they only need a compensation of 8%. We believe that these results are crucial for the investigation of the overconsumption of housing seen in the elderly data.

In some situations, it can be hard to determine the exact right amount of housing for retirees due to the complexities of financial planning and limitations in knowledge. Therefore, if a retiree is unsure about their optimal housing level, it’s better to err on the side of overestimating rather than underestimating it to ensure their well-being.

Many retirees stay in the same house they lived in before retiring. This is a positive deviation since retirees need smaller housing as the children have already moved out.

Changing a house can be expensive for retirees, both emotionally and financially. Besides the emotional attachment, there are costs involved in buying a new house and selling the old one. Our model does not consider these fees, but they could indirectly represent the complexity of decision-making. By staying in the same house during retirement, retirees avoid these fees. If we include these fees in our analysis, the welfare cost would be even lower, further supporting our findings.

We find that the impact of suboptimal housing decisions is less than that of suboptimal consumption decisions. This suggests that making optimal choices about consumption is more crucial than making optimal housing decisions. This is intuitive since consumption has a higher weight in an individual’s utility.

The utility cost of suboptimal housing is nearly identical under both *RU* and *SU*. This result is also confirmed by our analytical results which are presented after Proposition 3.

We want to clarify a possible misunderstanding. Our assumption is that a person buys a house at the beginning of retirement and does not move from that house until he dies. While it’s intuitive that a retiree might sell their house and move to a smaller one at the start of retirement, using the extra funds for other needs, most elderly individuals actually continue to live in the houses they bought before retiring. We analyze this decision of staying in the same, potentially oversized house as a suboptimal choice with a positive deviation, rather than analyzing the decision of staying in the same house as a suboptimal housing decision.

## 7 Sensitivity analysis

We test our model with different parameter values within the standard range to see how welfare costs vary. By changing one parameter at a time, we could understand how sensitive our results are to each parameter.

First, we vary the relative risk aversion (*RRA*) parameter to see how it affects welfare costs. As *RRA* increases (as *α* decreases), suboptimal stock holdings become less costly, while suboptimal bond holdings become costlier. For example, when *RRA* is increased from 3 to 5, the necessary compensation for a -50% deviation in stocks decreases from 0.95% to 0.6%. However, across the standard range of *RRA* values, we get low welfare losses for suboptimal stock and bond holdings. This relationship is intuitive: as retirees become more risk averse, stock decisions become less important, and bond decisions become more important for welfare.

Changes in the risk attitude (*RRA*) don’t affect the costs of suboptimal consumption or housing. Risk attitude only influences how people distribute their liquid savings, not the amount they save. Also, the welfare losses from suboptimal stock and bond holdings are consistently lower than those from suboptimal consumption and housing. This reaffirms our conclusion that “how people save is more crucial than where they invest.”

Second, we find that welfare costs are not sensitive to the discount factor (*β*). That is to say, our results are robust across different values of *β*. Third, we explore how the welfare costs change with the weight of housing in the utility, *ψ*. As *ψ* increases, only suboptimal housing becomes costlier. For example, the compensation needed for a 60% underinvestment in housing rises from 8% to 12% when *ψ* increases from 0.2 to 0.3. Similarly, the compensation for a 60% overinvestment in housing increases from 4% to 7%. Underinvestments remain costlier regardless of *ψ*.

As *ψ* increases, housing decisions become more important and consumption decisions become less consequential. For instance, the required compensation for a 60% underconsumption decreases from 39% to 33% when *ψ* increases from 0.2 to 0.3. Similarly, the compensation for a 60% overconsumption decreases from 24% to 19%. This change in *ψ* doesn’t alter the asymmetry in consumption: underconsumption remains costlier than overconsumption.

The welfare costs of suboptimal stock and bond holdings decrease slightly with higher *ψ*. This implies that as housing becomes more important, savings in liquid assets, bonds and stocks, become slightly less important. However, the parameter *ψ* does not alter our finding that negative deviations are costlier than positive deviations of the same degree in housing.

Fourth, welfare loss of suboptimal stock holdings decreases while that of suboptimal bond holdings increases dramatically with higher interest rate (*r*). To exemplify, the required compensation for a negative 50% deviation in stocks decreases from 1% to 0.1% when *r* is increased from 2% to 5%. Interest rate is the opportunity cost of investing in stocks. As *r* increases, bonds become more attractive relative to stocks. This shift explains why the welfare costs of suboptimal stock holdings decrease notably.

Changes in the interest rate (*r*) don’t affect the welfare costs of suboptimal housing or suboptimal consumption. Instead, *r* alters the mix of liquid savings but not the overall amount saved. Assuming a low value for *r*, increasing it would only reinforce our finding that suboptimal stock holdings have a low cost. Therefore, our conclusions about the low cost of suboptimal stock holdings and the higher cost of suboptimal bond holdings compared to stocks remain valid with higher values of *r*. In summary, our results remain consistent across different *r* values.

Some other researchers set the elasticity of intertemporal substitution (*EIS*) at values lower than 0.7, such as 0.05, 0.1, 0.25, 0.3, and 0.4. These lower values lead to higher welfare losses for suboptimal consumption, confirming our finding that suboptimal consumption has the highest welfare cost among all suboptimal cases. However, the welfare loss of suboptimal housing remains the same regardless of changes in *EIS*. Additionally, the welfare losses of both suboptimal bond and stock holdings only slightly increase with a lower *EIS*. This suggests that our main findings hold across various commonly used values of *EIS*.

To put in a nutshell, the costs of suboptimal consumption and housing are not influenced by changes in *RRA* and *r*. The time discount factor does not affect any of these costs. The costs of suboptimal stock and bond holdings are are only slightly affected by changes in the elasticity of intertemporal substitution (*EIS*) and the weight of housing.

Our findings are robust across different parameter values and utility specifications. For example, we consistently find low welfare costs for suboptimal stock and bond holdings regardless of the utility setting. We test our model with various parameter values and find consistently low welfare losses for suboptimal liquid investments. This reinforces our main result regarding the composition of liquid savings.

Some might question the value we used for the *RRA* parameter. While our choice falls within the standard range, there are some other studies which use higher values for *α* (equivalent to lower *RRA*). A higher *α* increases the welfare loss of suboptimal stock holdings. However, we still find low welfare costs.

The welfare costs of suboptimal consumption and housing are nearly identical under both *RU* and *SU*. This means that our findings regarding housing and consumption remain consistent regardless of the utility specification. Additionally, we find low welfare costs for suboptimal liquid investments under both utility specifications.

## 8 Policy implications

As we stated before, we find that the costliest elderly suboptimal decisions are consumption, housing and saving in a decreasing order. A comprehensive policy that improves the financial well-being of the elderly, helping them make better decisions and reduce the associated welfare cost could be beneficial. For example, the government could provide free or subsidized financial education for the elderly, focusing on budgeting, saving, and managing consumption. This could include workshops, or one-on-one counseling offered through community centers, libraries, or online platforms.

A certified expert may have better tools than a senior citizen with limited financial literacy. Ensuring that affordable and trustworthy financial advice is available to the elderly can help them make better decisions. The government could place mandatory financial education courses in the curriculums of high schools or universities. The government could ensure that financial services are accessible and user-friendly for the elderly, including online banking, bill payment services, and investment platforms.

We find that the decision of how much to consume vs save is more important than the composition of saving. Policy makers could educate retirees on the importance of balancing consumption with savings, budgeting in retirement, coping with market volatility and the risks associated with outliving their savings. Experts could create software tools that can be integrated into pension fund websites or offered as standalone apps. These tools would use algorithms to suggest spending recommendations based on market conditions and personal savings levels. The government could encourage savings among the elderly by offering tax incentives for savings vehicles or by creating government-backed savings programs specifically designed for the elderly with favorable interest rates.

As mentioned before, we find a modest (3.7%) welfare cost for zero stock holding which be regarded as negligible or zero if we also include rare-disaster cost of equities, transaction, and complexity costs. This suggests that retirees might not be significantly worse off by not holding stocks in their portfolios. In this case, a policy intervention might focus less on encouraging stock investment and more on ensuring overall financial security and literacy for retirees. A potential policy to address this could offer comprehensive financial education programs for the elderly covering investment options other than stocks.

We find a welfare cost of almost 7% for the case of zero bond holdings. If the welfare cost of zero bond holdings for retirees is higher than that of zero stock holdings, it suggests that bonds play a more critical role in retirees’ portfolios, likely due to their stability and income-generating potential. In this case, a policy could be designed to encourage retirees to include bonds in their investment portfolios. The government may offer tax incentives for retirees who invest in bonds, such as tax exemptions or credits on interest income earned from bonds, to make them more attractive as an investment option. The program may simplify access to bond investments for retirees by offering easy-to-understand bond funds or government savings bonds that are specifically designed for retirement income.

We also find that under-consumption is costlier than the same degree of over-consumption. Government may implement a policy for older adults, particularly those who could be at risk of under-consumption due to inadequate savings or fixed incomes. Eligible individuals would receive a guaranteed minimum income, which would be set at a level sufficient to cover basic living expenses, including food and other necessities. The government can give tax returns to the elderly for basic consumption items. Moreover, the government could strengthen consumer protection laws to prevent financial exploitation and fraud targeting the elderly, which can erode their savings and affect their consumption.

We find that in housing the required compensation for a positive deviation is markedly lower than that for the same degree of negative deviation. The government may assist the senior citizens in maintaining their current housing instead of downsizing. One possible policy approach could be the creation of a compensation fund for tenants who experience significant negative deviations in their housing quality due to factors beyond their control (e.g., natural disasters, landlord negligence). The government could invest in the development of affordable housing options to reduce the likelihood and impact of negative deviations in housing, thereby addressing the welfare loss associated with the higher required compensation for negative deviations compared to positive ones.

## 9 Conclusion

We have developed a realistic framework to study retiree decisions under *RU* with housing. We have quantitatively assessed welfare losses for deviations from optimal decisions, analyzing three cases analytically and three cases numerically.

We have discovered that suboptimal equity investments have moderate welfare costs. In contrast, suboptimal consumption decisions can be very costly. This suggests that making optimal decisions regarding consumption and savings is crucial, regardless of whether one saves through bonds or stocks. While suboptimal housing choices are less costly than suboptimal consumption decisions, they are still costlier than suboptimal bond and stock investments.

Numerous retirees reside in homes larger than their optimal size, representing a positive deviation in housing. Interestingly, we find that negative deviations in housing are costlier than positive deviations of the same magnitude.

The welfare costs of suboptimal consumption and housing are the same under both *RU* and *SU*. Additionally, we find that welfare costs of suboptimal bond and stock holdings are low in both scenarios.

This study is among the first to analyze the welfare costs of suboptimal allocations for retirees under the *RU* setting with housing. The potential inclusion of frictions, such as stock market participation fees or housing adjustment costs, does not hinder the clarity of our conclusions. In fact, these frictions would likely strengthen our main findings. Our sensitivity analysis demonstrates that our results are robust and not contingent on our specific parameter choices or utility function.

There are some other factors that affect the asset allocations of retirees, such as variable bequest motives and health risk. A strong bequest motive or the presence of health risks might reduce the negative impact of under-consumption, especially in the early years of retirement. Additionally, the inclusion of bequest motives could decrease the costs associated with over-investment in housing. Thus, the addition of bequest motive will strengthen our result about the housing puzzle of the elderly. However, the analytical solution could not be obtained with the inclusion of these factors. Future research could explore extending the model to incorporate these factors and further enhance our understanding of retirees’ decisions.

Our model could be extended by frictions and fees regarding the purchase of assets and/or habit formation regarding housing and cost of decision-making complexity subject to the level of financial literacy. We find low welfare costs for many suboptimal puzzling retiree scenarios. These costs will be even lower with the addition of some frictions and fees.

While we focus on the retirement horizon in our model, future research could extend the analysis to cover the entire life-cycle. However, obtaining analytical solutions for the periods before retirement may be challenging. Another potential direction for future research is to model housing as an asset, which could provide further insights into retirees’ financial decisions.

We focus on analyzing the effects of individual deviations, such as only deviating in stock investments, without considering how these choices might interact. Future research could explore how different choices interact and affect overall welfare. Additionally, our analysis could be extended to investigate other potential suboptimal behaviors beyond those considered in this study.

While we focus on suboptimal bond and stock investments, future studies might address suboptimal investments in Islamic Financial Market ([71] studies the effect of Islamic Financial Market in economic growth) such as sukuk and Sharia stocks.

## Supporting information

S1 Appendix Explanations about the equations 11-13 and the variables. (PDF)
