# Supplementary material for: Welfare costs of suboptimal retiree decisions
Source: PLoS One. 2024 Aug 27;19(8):e0307379. doi: 10.1371/journal.pone.0307379 (PMC11349116; doi:10.1371/journal.pone.0307379)
Supplement: S1 Appendix — (PDF) [file pone.0307379.s001.pdf]

1 S1 Appendix

1.1 Explanations about the equations 11-13 and the variables introduced

1.1.1 Equation 11

$b_t = \frac{l}{1+l+m_t(d+Rl)}$

Equation 11 addresses the allocation of current liquid assets into bonds, which are considered highly secure investment vehicles. A reduction in the denominator's value would imply a higher proportion of wealth should be allocated to bonds to enhance safety and meet immediate financial needs.

1.1.2 Equation 12

$c_t = \frac{m_t(d+Rl)}{1+l+m_t(d+Rl)}$

Equation 12 delineates how to allocate wealth for immediate consumption, conceptualizing total wealth as a pie. This equation assists in determining the size of the slice that can be consumed now, ensuring future needs are not compromised. The term m_t serves as a personal benchmark that reflects the importance of saving for unforeseen circumstances, acting as a discount factor that modifies future utility derived from consumption. The component $(d + Rl)$ assesses the volatility or risk of the stock market. Here, d represents the risk of significant market downturns, while Rl symbolizes the protective measures or safer investments that buffer against potential financial losses. A lower concern for future expenses or a favorable risk assessment allows for a larger immediate consumption from one's wealth and enjoying a bigger slice of pie today.

1.1.3 Equation 13

$\pi_t = \frac{1}{1+l+m_t(d+Rl)}$

Equation 13 serves as a financial recipe or guide for determining the portion of savings that should be allocated to the risky or volatile stock market. It essentially asks, "What proportion of savings should be risked on stocks?" The numerator is just like saying, "Let's start with everything I've got." The variable "l" provides an adjustment based on an individual's risk tolerance. Those who are comfortable with the market's fluctuations might allocate a larger share to stocks. On the other hand, individuals prioritizing future consumption and financial security might want to be more cautious with how much invested in stocks right now. Moreover, a high value of "d" signals a significant risk of loss, which could encourage to think twice about going heavy into stocks. investments.

1.1.4 The introduced variables

$k = [\frac{(d-R)q}{p(R-u)}]^{\frac{1}{(1-\psi)\alpha-1}}$, $l = \frac{kd-u}{R(1-k)}$

"k" is essentially assessing the attractiveness of a stock investment by comparing the expected loss against the expected gain, all while considering the person's unique financial comfort zone and spending habits.

"l" can be seen as a sort of financial balancing scale. It's a metric that tells how to balance between securing a baseline return and venturing out for potentially higher gains, depending on the person's risk tolerance and investment goals.

$g_t = p(b_tR + u\pi_t)^{(1-\psi)\alpha}$

This equation reflects the expected increase in happiness (or utility) from the portfolio when things go well in the stock market. While the component b_tR reflects the expected return from bonds, $u\pi_t$ gives the expected growth of the stock portion of the portfolio if the stock market goes up. The utility is weighed by p, the probability of the stock market going up. The last term in the equation adjusts the growth by the utility derived from non-housing consumption and the individual's risk aversion.

$z_t = q(b_tR + d\pi_t)^{(1-\psi)\alpha}$

The equation for z_t mirrors the equation for g_t for the downside risk. So, z_t tells how much unhappiness (or loss in utility) a person might expect when things don't go well in the stock market. Th component b_tR is consistent in both up and down scenarios. The second component $d\pi_t$ reflects the expected decrease in the stock portion of the portfolio if the stock market goes down. The utility loss is weighed by q, the probability of the stock market going down.

$u_t = (c_t^{(1-\psi)\phi} + \beta[g_t + z_t]^{\phi/\alpha})^{1/\phi}$

The equation for u_t is a kind of happiness recipe considering the immediate pleasure derived from current expenditures, such as purchasing a cup of coffee or a movie ticket (represented by c_t) and alongside the discounted value of expected future happiness from investment gains (g_t) and losses(z_t). The β part serves to moderate the weight assigned to future happiness, acknowledging that it is not as immediate as current enjoyment. The adjustment part (the part with the ϕ and α symbols) ensure this recipe respects or accomodates individual risk preferences and the balance between immediate gratification and future well-being So, u_t represents a composite measure that balances current enjoyment with strategic planning for future contentment.

$\beta_t = \beta\rho_t u_{t+1}^{\phi}$

β_t represents an adjusted metric for valuing future satisfaction, incorporating personal preferences such as the flexibility in timing life's pleasures(represented by ϕ. It is considering, "Is it okay to skip the vacation now and have it later?"). Additionally, it considers probabilistic factors that address the certainty of being able to enjoy future savings, which is captured by ρ_t. This factor contemplates the potential that one might not be present to experience the benefits of their savings. The expected level of happiness from future expenditures is represented by u_{t+1} which might involve decisions such as choosing between a big vacation or regular cozy dinners at home. A higher β_t suggests a greater propensity to save now for potentially better experiences later, reflecting a future-oriented disposition. Conversely, a lower β_t indicates a preference for immediate gratification, leading to a decision to spend more in the present rather than saving.

$m_t = \{\beta_t[pk^{(1-\psi)\alpha} + q]^{\frac{\phi-\alpha}{\alpha}}(puk^{(1-\psi)\alpha-1} + qd)\}^{\frac{1}{(1-\psi)\phi-1}}$

The equation for m_t calculates the present value of future utility derived from consumption, serving as a financial planning tool. It assesses the utility one might expect from future consumption based on preferences for housing and other goods, patience levels, and aversion to risk, while also accounting for the volatility of the stock market. The variable p adjusts for the chance that things might go well in the stock market and q assesses the potential for adverse stock market conditions. The parameters α and ψ are personal adjustments that reflect an individual's willingness to wait for future rewards and their tolerance for risk. So, m_t is basically a way to translate future happiness from money into today's terms, similar to considering whether the immediate enjoyment of purchasing a new gadget outweighs the opportunity cost of not having that money available for future use.
